# Elastic distortion determining conduction in BiFeO_3_ phase boundaries†

**DOI:** 10.1039/d0ra04358c

**Published:** 2020-07-27

**Authors:** Kristina M. Holsgrove, Martial Duchamp, M. Sergio Moreno, Nicolas Bernier, Aaron B. Naden, Joseph G. M. Guy, Niall Browne, Arunava Gupta, J. Marty Gregg, Amit Kumar, Miryam Arredondo

**Affiliations:** School of Mathematics and Physics, Queen's University Belfast UK kholsgrove04@qub.ac.uk m.arredondo@qub.ac.uk; Ernst-Ruska Centre for Microscopy Juelich Germany; Nanyang Technological University Singapore; Bariloche Atomic Centre San Carlos de Bariloche Argentina; Univ. Grenoble-Alpes, CEA, Leti France; University of St. Andrews UK; Center for Materials and Information Technology, University of Alabama USA

## Abstract

It is now well-established that boundaries separating tetragonal-like (T) and rhombohedral-like (R) phases in BiFeO_3_ thin films can show enhanced electrical conductivity. However, the origin of this conductivity remains elusive. Here, we study mixed-phase BiFeO_3_ thin films, where local populations of T and R can be readily altered using stress and electric fields. We observe that phase boundary electrical conductivity in regions which have undergone stress-writing is significantly greater than in the virgin microstructure. We use high-end electron microscopy techniques to identify key differences between the R–T boundaries present in stress-written and as-grown microstructures, to gain a better understanding of the mechanism responsible for electrical conduction. We find that point defects (and associated mixed valence states) are present in both electrically conducting and non-conducting regions; crucially, in both cases, the spatial distribution of defects is relatively homogeneous: there is no evidence of phase boundary defect aggregation. Atomic resolution imaging reveals that the only significant difference between non-conducting and conducting boundaries is the elastic distortion evident – detailed analysis of localised crystallography shows that the strain accommodation across the R–T boundaries is much more extensive in stress-written than in as-grown microstructures; this has a substantial effect on the straightening of local bonds within regions seen to electrically conduct. This work therefore offers distinct evidence that the elastic distortion is more important than point defect accumulation in determining the phase boundary conduction properties in mixed-phase BiFeO_3_.

## Abstract 2

The localized crystallography of conducting and non-conducting phase boundaries in mixed-phase BiFeO_3_ is directly compared using scanning transmission electron microscopy techniques.

## Graphical abstract

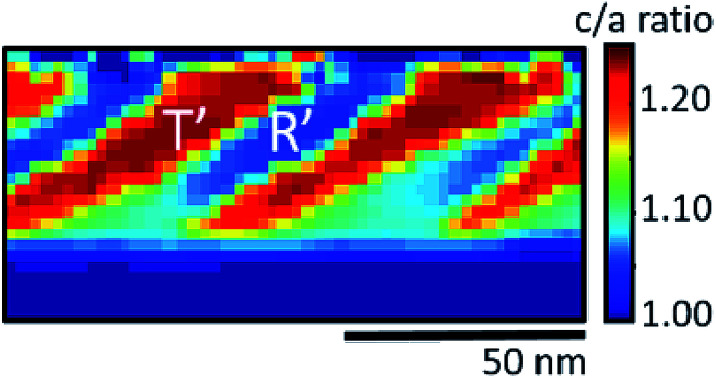

The complexity of electrical conductivity in domain walls in BiFeO_3_ (and in ferroics in general) is as multifaceted as ever. Various influences such as point defect accumulation, octahedral rotations, magnetic interactions and electrostatic discontinuities are thought to be possible mechanisms at play,^1–8^ either alone or in combination. The research area of domain wall conductivity is currently flourishing and the view that domain walls offer exciting prospects in terms of engineering systems in which the domain walls act as distinct identities to the domains which they separate is now widely accepted. We believe that it is pertinent timing to address a lack of experimental investigations providing meaningful direct comparison of the localised crystallography and defect structure responsible for observed enhanced electrical conductivity. This study is stimulated by the interesting discoveries of conductive phase boundaries, specifically, in mixed-phase BiFeO_3_.^9,10^ By tuning the local populations of the tetragonal-like (T) and rhombohedral-like (R) phases in BiFeO_3_ thin films *via* electric and stress fields, we demonstrate that electrical conductivity along phase boundaries is significantly greater after stress-writing. We probe the key crystallographic differences between the R–T boundaries created *via* stress, compared to those already present in the as-grown microstructures, to disentangle the mechanism determining electrical conduction in mixed-phase BiFeO_3_.

The growth of BiFeO_3_ on substrates enforcing a large in-plane compressive strain drives the formation of monoclinic phases that are approximately rhombohedral (R) and tetragonal (T). Similar to materials such as PbZr_0.53_Ti_0.47_O_3_ that straddle a morphotropic phase boundary, highly strained BiFeO_3_ can readily undergo phase transitions between the R and T phases (and *vice versa*). The high-strain T phase exhibits a tetragonal-like symmetry (almost *P*4*mm*) with a *c*/*a* ratio of ∼1.2; the Fe displacement towards one of the apical oxygens along [001]_pc_ results in fivefold oxygen coordinated Fe, and an enhanced polarisation roughly 1.5 times that of the bulk single crystal.^7,11^ The R phase, on the other hand, resembles the rhombohedral bulk phase (almost *R*3*c*), where the Fe is octahedrally coordinated, with a ferroelectric distortion along the pseudocubic [111]_pc_ axis, and antiferrodistortive rotations of the FeO_6_ octahedra around [111]_pc_ occur. The crystal structure and misfit strain associated with the native (as-grown) R and T phases is reported elsewhere, both theoretically^12–15^ and experimentally,^6,7,16–21^ making it well-known that the ferroelectric and the antiferrodistortive degrees of freedom in mixed-phase BiFeO_3_ set it apart from other typical perovskites. Notably, despite the ample evidence provided on phase reversal and characterisation of the as-grown phases, most of the literature (especially regarding electric field cycling of the mixed-phase state) has been primarily concerned with X-ray diffraction (XRD) *i.e.* global measurements that will not necessarily pick up on the more subtle, atomic-scale aspects of structure local to the phase boundaries. The importance of the study described herein resides in the uniqueness of creating microstructures such that both the as-grown and stress-induced R–T phase boundaries can be included within one single cross-sectional transmission electron microscopy (TEM) lamella; this gives the best possible scenario to allow meaningful direct comparison of the localised crystallography and defect structure responsible for the observed enhanced electrical conductivity found at stress-induced phase boundaries.

## Demonstration of enhanced conductivity at stress-induced BiFeO_3_ phase boundaries

**Fig. 1:**
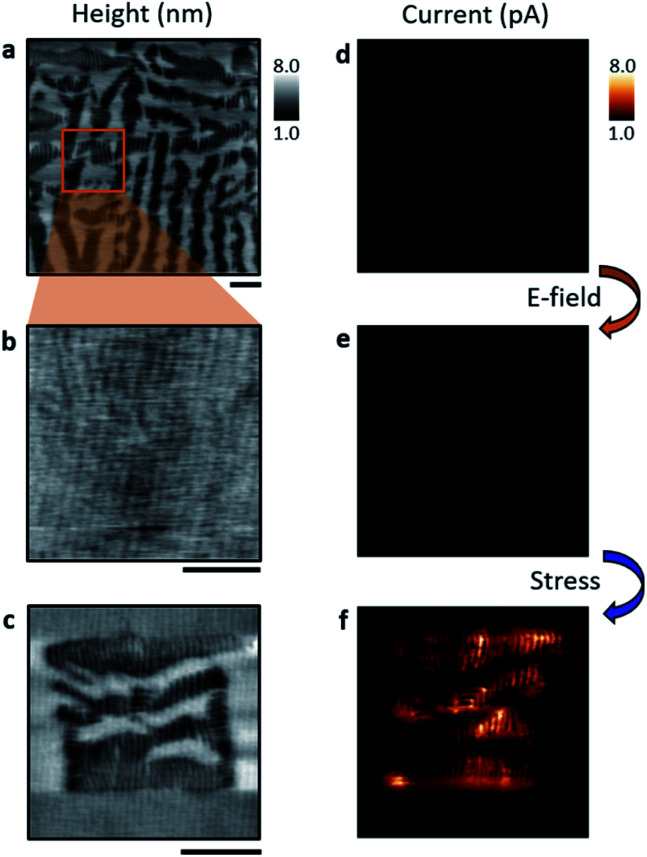
Stress-induced mixed-phase demonstrating increased conduction. Topography of (a) native mixed-phase microstructure, (b) after application of 30 V into a T-phase within the area marked by a box in (a), and (c) after subsequent tip-application of approximately 1200 nN into a newly-formed mixed-phase microstructure. Corresponding c-AFM measurements of the initial native (d), electrically-written (e) and stress-induced mixed-phase (f) regions showing increased currents in the order of pA in the stress-written region. Scale bars are 1 μm.

Fig. 1 demonstrates how electric field and stress-writing can be employed to alter the relative phase population in mixed-phase BiFeO_3_ films (∼35 nm thickness), grown epitaxially atop a LaAlO_3_ substrate with a 5 nm La(Sr,Co)O_3_ bottom electrode, corresponding to a misfit strain of −4.4% relative to single crystal BiFeO_3_.^12,22^ In a 〈001〉_C_-oriented film, an out-of-plane electric field, applied through an atomic force microscope (AFM) tip, leads to conversion of R- to T-phase, due to polarisation rotation from 〈111〉_C_ to 〈001〉_C_. Conversely, uniaxial stress, also applied using an AFM tip, leads to T-to-R conversion by 〈001〉_C_ to 〈111〉_C_ polar rotation.^22–24^ To illustrate this, a region of the film with native R and T phases (Fig. 1(a)) was switched electrically into a region of pure T-state (Fig. 1(b)), using a 30 V tip potential. Following this, stress was applied within the T-phase region (Fig. 1(c)), through scanning of the AFM tip with approximately 1200 nN (∼0.61 GPa) to the sample surface, resulting in the injection of R-phase needles and the recreation of a mixed-phase microstructure. Conducting AFM (c-AFM) measurements (using DC voltage below the ferroelectric coercive field) reveal negligible conduction in the as-grown mixed-phase and electrical bias written T-phase (see Fig. 1(d) and (e) respectively). The stress-induced mixed-phase microstructure, on the other hand, shows significant conduction (Fig. 1(f)). In agreement with other reports,^9^ the highest currents are observed where stress-induced R–T boundaries (hereon denoted as R′–T′ boundaries to distinguish them from their as-grown R–T counterparts) are present and are found to be stable over a measured period of several days.

## Spatially resolved distribution of oxidation states and point defects

It has been widely assumed that oxygen vacancies and associated mixed valence states in Fe are responsible for phase boundary electrical conductivity.^2,9,25–28^ To identify the distribution of oxidation states and presence of point defects, a cross-section was prepared using focused ion beam (FIB) milling. A region containing both as-grown R–T and stress-induced R′–T′ microstructures was selected, allowing for direct comparison of insulating and electrically conducting phase boundaries (see ESI† for more details on specimen preparation).

**Fig. 2:**
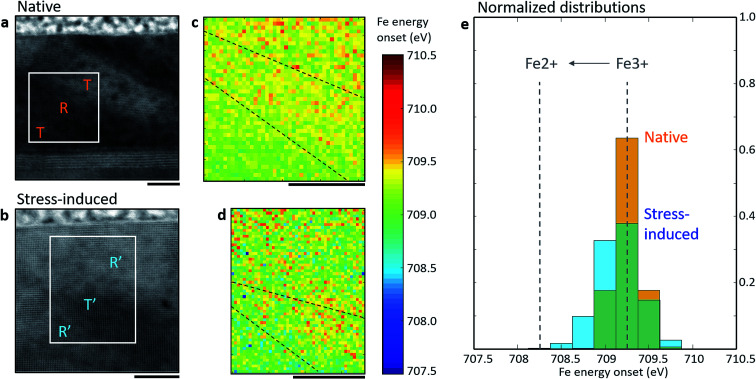
Fe oxidation state distribution in the native and stress-induced phases. ADF-STEM overview of a (a) native and (b) stress-written region. Fe edge onset maps for the (c) native and (d) stress-written areas enclosed by the white boxes in (a) and (b) respectively. (e) Normalised histograms for the Fe edge onset energies measured in the native and stress-written regions. Ideal energy difference between Fe^3+^ and Fe^2+^ oxidation state labelled with grey dotted lines. Scale bars are 10 nm.

To probe the local distribution of Fe oxidation states/oxygen vacancies, we employed electron energy loss spectroscopy (EELS), as shown in Fig. 2. Spectrum images were acquired across the entirety of both as-grown and stress-written regions, ensuring that all the possible FeO_6_ environments were measured. Scanning transmission electron microscopy (STEM) annular-dark field (ADF) images of native and stress-written regions are shown in Fig. 2(a) and (b), respectively. The white box annotations mark the regions where spectrum images were acquired and Fig. 2(c) and (d) show maps of the Fe L-edge onset which is well known to increase monotonically with increased Fe oxidation state,^29^ with Fe^2+^ occurring at ∼1 eV lower than Fe^3+^, as shown in the histogram in Fig. 2(e). From the histogram and maps, it is clear that the oxidation state is distributed around Fe^3+^ and this distribution is relatively homogeneous in both native and stress-written regions, it is not localised to the mixed-phase boundaries. In other words, a homogenous distribution of point defects (O vacancies) is observed which is not consistent with the model of phase boundary mixed oxidation states as the primary mechanism for electrical conduction.

## Strain state and effects on local bonding

**Fig. 3:**
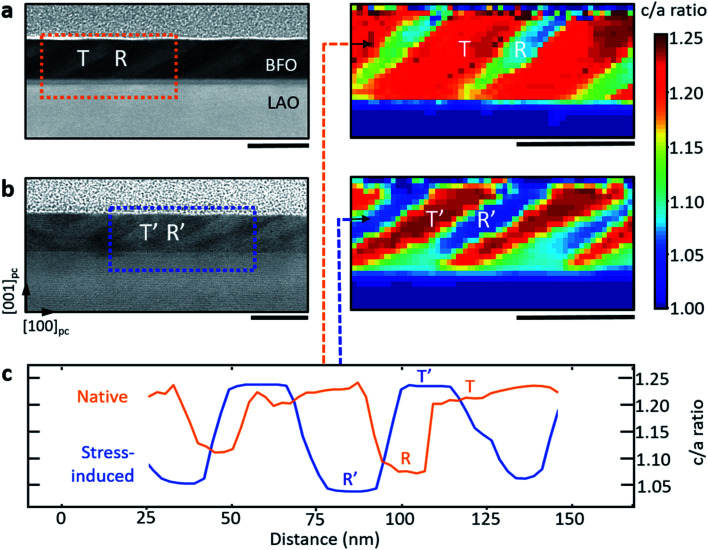
Tetragonality maps of (a) native and (b) stress-induced phases in BiFeO_3_. ADF-STEM images acquired along the [010]_pc_ zone axis (left column) and NBED maps (right column) of native and stress-written regions. Line profiles (c) of the *c*/*a* ratio for the native and stress-written regions extracted from the horizontal row of pixels marked by an arrow in the NBED maps. Scale bars are 50 nm.

The functional properties of perovskite oxides are known to be especially sensitive to the materials' crystallography. We therefore performed combined annular-dark field STEM (ADF-STEM) and nano-beam electron diffraction (NBED) studies of the as-grown and stress-induced mixed-phase microstructures. Fig. 3 shows ADF-STEM images (left column) and NBED tetragonality maps (right column) of (a) native and (b) stress-induced regions. We observe that there is a variation in the depth of blue and red colouration in the R–T compared to R′–T′ phases. Absolute variations in *c*/*a* ratio are clearly present; these are further illustrated in the line profiles in Fig. 3(c) revealing typical native *c*/*a* ratios of 1.08 for R and 1.22 for T, which are consistent with previous experimental studies,^7,12,16^ and importantly suggest little in the way of thin film relaxation during sample preparation. The tetragonality of the phases, produced after stress-writing, show averaged *c*/*a* ratios of 1.05 for R′ and 1.24 for T′, suggesting newly-strained unit cells (with associated volume variation): lower strained R′ phases and higher strained T′ phases.

**Fig. 4:**
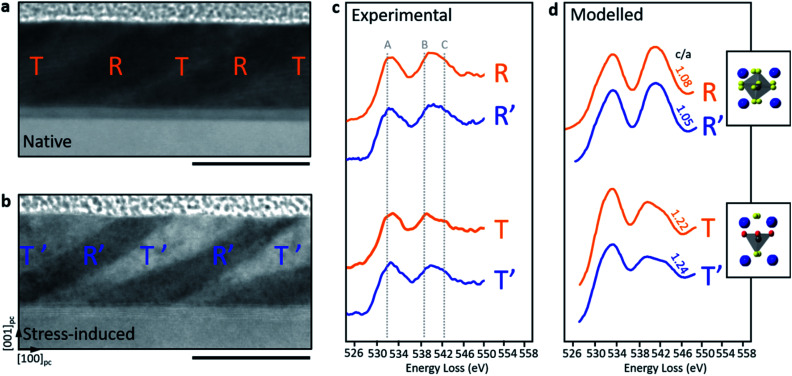
Electronic structural signature of native and stress-induced phases. ADF-STEM images of representative (a) native and (b) stress-written regions. Experimentally measured O–K edge (c) for the native R and T phases and stress-induced R′ and T′ phases. (d) Calculated O–K edge for the corresponding native and stress-induced phases using BiFeO_3_ crystal structures matching the *c*/*a* ratios from the NBED data obtained in Fig. 3. BiFeO_3_ unit cell schematics corresponding to the R and T phases are also included, blue, grey and yellow spheres represent Bi, Fe and oxygen atoms, respectively. For the T-phase models, the equatorial O(2) oxygen atoms are shown in red to differentiate them from the yellow apical O(1) oxygen atoms. Scale bars are 50 nm.

Altered tetragonality can be anticipated to be accompanied by changes to the electronic structure of the material through adjustments in O bonding/coordination. This can be observed *via* the fine structure of the O–K EELS edge, as shown in Fig. 4. The most significant differences between the R and T phases are known to arise in the O–K edge sub-peaks labelled ‘B’ and ‘C’ in Fig. 4(c). These peaks refer to the outermost Fe electrons, originating from hybridized O 2p states with Fe 4sp and covalently bonded to Bi states.^14,17,21^ Importantly, the R phase has 8-fold oxygen coordination with respect to Fe while the native T phase has 5-fold coordination. This change in the oxygen coordination is manifested in a reduction of peak ‘C’ in the T phase.^17^ If we compare T–T′ and R–R′ phases for the as-grown *vs.* stress-induced O–K edges, it is noticeable that the variation in tetragonality (*c*/*a* ratio) contributes to subtle spectral alterations in the ‘B’ and ‘C’ peaks (at ∼539 eV and ∼543 eV). These spectral distinctions have been verified by calculating the O–K edge of BiFeO_3_ crystal structures created by A. Hatt and N. A. Spaldin^12^ ranging from a highly-strained regime (6.4% compressive strain) to an extremely low strained regime (0.9% compressive strain) using FEFF, which uses an *ab initio* self-consistent real space multiple scattering approach to replicate electronic structure under matching experimental conditions.^30^Fig. 4(d) displays the calculated O–K edges corresponding to the relevant crystal structures (*i.e.* matching the *c*/*a* ratios to the experimental data of Fig. 3).

**Fig. 5:**
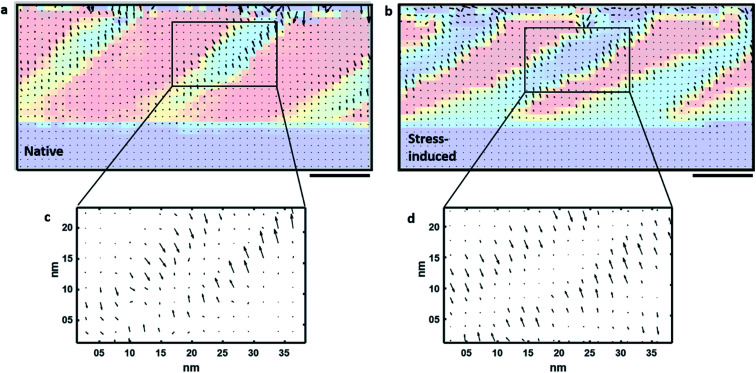
Mapping phase boundary elastic distortion. Strain gradient vectors for (a) as-grown and (b) stress-induced microstructures with *c*/*a* ratio maps from Fig. 3 under laid. (c and d) Higher magnification extracts of strain gradient vectors alone, showing comparable local distortion magnitudes, but the thickness of the distorted regions is much greater in the stress-induced microstructures. We note that stray field arrows originating from the top of the thin film (where the Pt bar is located) have not been removed from these figures but should be considered with caution. Scale bars are 20 nm.

If the alterations in the unit cells in the R′ and T′ phases of the stress-written region are greater than those seen in the as-grown R and T phases, then the phase boundaries must accommodate more significant elastic change. Such strain gradients have recently been demonstrated at phase boundaries, having associated effects on the local photoelectric properties in strained BiFeO_3_ thin films, with giant strain manifested at the R–T boundaries giving rise to photoconduction.^10^ By carefully analysing local variations in lattice parameters (*via* NBED) we have mapped the in-plane (*ε*_*xx*_) and out-of-plane (*ε*_*yy*_) strain in the as-grown and stress-induced microstructures. From these measurements, we calculated the strain gradient vectors. Fig. 5(a) and (b) show the strain gradient vectors mapped across the as-grown and stress-induced regions presented earlier in Fig. 3; the *c*/*a* ratio maps are under-laid behind the equivalent strain gradient vector maps for comparison. We observe that the strain gradient magnitudes (size of arrows) are similar, but the regions over which they exist are much more extensive in the stress-induced regions; this is perhaps most obvious in the higher magnification maps (Fig. 5(c) and (d)). This is important as it means that larger regions of elastic distortion exist in the stress-induced microstructures compared to those of the virgin film. Local distortion is comparable, but the spatial thickness of the distorted regions is much greater.

A more extensive elastic distortion is expected to have some effect on the local bond angles in the R′–T′ phase boundary regions. We note that the idea of octahedral bond angle straightening linking to increased conductivity in domain walls was first hypothesised by G. Catalan and J. Scott in their review paper of BiFeO_3_ in 2009.^5^ This idea was inspired by previous work on the perovskite nickelates,^31–33^ which demonstrated that straightening the Ni–O–Ni angle (by increasing rare-earth-radius, external pressure, or temperature) increases the orbital overlap, reduces the band gap and stabilises the metallic state.^34^ With further evidence, Catalan pointed out that the correlation between Fe–O–Fe straightening and band gap reduction in BiFeO_3_ may be fortuitous and not necessarily the cause of increased conductivity.^35^ Recent theoretical work by Chen *et al.* also suggests that the straightening of Fe–O–Fe does not have any obvious correlation with bandgap reduction.^36^ Their study reveals that the domain wall electronic properties of BiFeO_3_ sensitively depend on the polarisation behaviour and the structural distortion at the domain wall centre. On the other hand, Han *et al.* recently reported a high resolution STEM study of BiFeO_3_ thin films grown on PrScO_3_ substrate with 180° conducting domain walls, where the [110]_pc_ projected Fe–O–Fe bond angles reveal a distinct decrease (*i.e.* buckling rather than straightening of the bond angle).^37^

To our knowledge, localised evidence of Fe–O–Fe bond angle straightening (or buckling) in mixed-phase BiFeO_3_ has not yet been observed experimentally. Direct measurement of bond angles is challenging in these systems since their large epitaxial strain necessitates a relatively large TEM lamella thickness to avoid thin foil relaxation effects. We found that to maintain the presence of R–T phase boundaries, the thickness of the FIB cross-section should not be reduced lower than ∼60 nm. In this scenario, the imaging and interpretation of light O atoms and heavy Bi and Fe atoms *via* combined HAADF and bright field (BF)-STEM techniques is difficult. Nevertheless, we provide HAADF and BF-STEM images in the ESI† where efforts have been made to measure local bond angles across conducting R′–T′ phase boundaries. Our observations and simulations (Fig. S4†) suggest phase contrast in Fe–O–Fe measurements and a buckling of the Bi–O–Bi bond angles across conductive phase boundaries. We propose that these local bond angle variations were induced by the extensive elastic distortion shown at phase boundaries exhibiting enhanced electrical conduction.

## Conclusions

We have used advanced microscopy techniques to study conducting phase boundaries in mixed-phase BiFeO_3_ samples, where local populations of T and R can be readily altered using stress and electric fields. We noticed that electrical conductivity along phase boundaries is significantly greater in the stress-induced microstructures. Detailed comparison of the R–T phase boundaries created by localised stress with those already present in the as-grown films was carried out to identify crystallographic differences, to better understand the mechanisms responsible for electrical conduction. We found that point defects (and associated mixed valence states) are present in both electrically conducting and non-conducting regions; significantly, in both cases, the spatial distribution of defects is relatively homogeneous: there is no evidence of phase boundary defect aggregation. Atomic resolution imaging revealed that the only significant difference between non-conducting and conducting phase boundary regions is the elastic distortion; the accommodation of distortion is much greater in stress-induced than in as-grown microstructures, having substantial effects on the local bonding angles. This work strongly suggests that the elastic distortion is more important than point defect accumulation in determining conducting properties in mixed-phase BiFeO_3_.

## Experimental methods

### Thin film processing

Epitaxial BiFeO_3_ thin films of thickness ∼35 nm were grown using the pulsed laser deposition technique on (001)-oriented LaAlO_3_ substrates with a 5 nm La(Sr,Co)O_3_ bottom electrode. Details of the sample preparation have been reported elsewhere.^38^

### Stress and electrical writing

The imaging of the topography as well as stress and electrical writing was carried out using a Veeco Dimension 3100 AFM system (with a Nanoscope IIIa controller) in conjunction with a Keithley 6517B electrometer. The mechanical writing was performed by increasing the deflection set-point to 8 V (*i.e.* the cantilever deflection voltage maintained by the atomic force microscope feed-back loop proportional to the applied force or stress) to a Pt-coated Si tip (Nanosensors PPP-EFM) while scanning. Determining the spring constant was made possible using a combination of force–distance curves and thermal tuning methods available on an MFP-3D Infinity AFM system, the spring constant was found to be 2.8 Nm^−1^. Each 1 V of deflection set point from the tip corresponds to a loading force of about 150 nN.

### Conducting atomic force microscopy measurements

Current measurements were acquired with a c-AFM application module (ORCA, Asymlum Research) by applying a +3.5 V DC bias to the bottom electrode under ambient conditions at room temperature, with all c-AFM measurements made immediately after electrical and mechanical switching.

### Electron microscopy

Cross-sectional lamellae were fabricated using standard focused ion beam (FIB) lift-out procedures on a FEI Helios NanoLab 460F1 FIB-SEM.^39^ STEM imaging and NBED were performed on a Cs corrected FEI Titan operated at 300 kV, with a convergence angle *α* = 0.5 mrad for NBED.^40^ Strain was quantified from NBED patterns using a dedicated script developed by CEA, Grenoble.^41^

### EELS simulations

Multiple-scattering calculations were performed to correlate the energy loss near edge spectral features in the oxygen and Fe edges with the experimental EELS data. The calculations were based in the FEFF9 code.^42,43^ Accurate and well converged muffin-tin potentials and electron densities were calculated in a self-consistent-field (SCF) procedure.^44^ See ESI† for calculations of density of states.

## Author contributions

K. M. H., M. A. and A. K. conceived of and designed the experiments. A. G. grew the studied films. K. M. H. and M. D. carried out the electron microscopy experiments. A. B. N. helped in the data analysis and interpretation. N. B. processed the NBED strain maps. M. S. M. performed the EELS simulations presented in the study. J. G. M. G. and N. B. carried out the atomic force microscopy experiments. K. M. H., A. B. N., M. A., A. K. and J. M. G. wrote the manuscript with input from all authors.

## Conflicts of interest

There are no conflicts to declare.

## Supplementary Material

RA-010-D0RA04358C-s001
