# Supplementary material for: Elastic distortion determining conduction in BiFeO3 phase boundaries
Source: RSC Adv. 2020 Jul 27;10(47):27954–60. doi: 10.1039/d0ra04358c (PMC9055675; doi:10.1039/d0ra04358c)
Supplement: RA-010-D0RA04358C-s001 [file RA-010-D0RA04358C-s001.pdf]

Electronic Supplementary Material (ESI) for RSC Advances.
This journal is © The Royal Society of Chemistry 2020

Supplemental Material: Elastic Distortion Determining Conduction in $BiFeO_3$ Phase Boundaries

Kristina M. Holsgrove*[a], Martial Duchamp [b,c], M. Sergio Moreno[d], Nicolas Bernier[e], Aaron B. Naden[a,f], Joseph Guy[a], Niall Browne[a], Arunava Gupta[g], J. Marty Gregg[a], Amit Kumar[a] and Miryam Arredondo[a]

[a]School of Mathematics and Physics, Queen's University Belfast, Belfast, UK
[b]Ernst-Ruska Centre for Microscopy, Juelich, Germany
[c]Nanyang Technological University, Singapore
[d]Bariloche Atomic Centre, San Carlos de Bariloche, Argentina
[e]University of Grenoble Alpes, Grenoble, France
[f]University of St. Andrews, UK
[g]Center for Materials and Information Technology, University of Alabama, USA

S1 - Experimental details of the specimen preparation

Typically, the polished lamella included: a region of as-grown (native) mixed phase R-T microstructures for reference, an electrically-written (T-state) region, and a region of stress-induced mixed phase R'-T' microstructure.

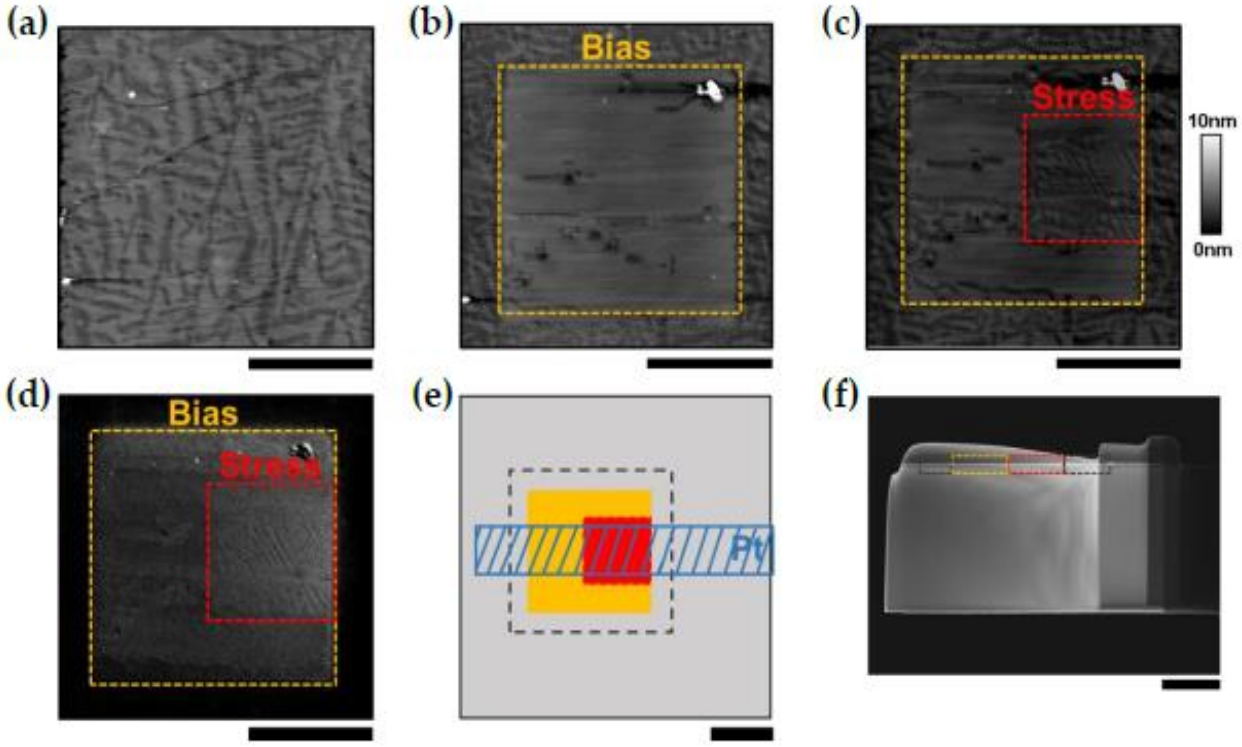

AFM topography of the native mixed-phase state (a), after application of -5V dc electric field (b) and after 800nN of tip-induced force (c). SEM image of the electrically-written and stress-written regions identified (d). Schematic of the Pt-strip position for the lamella preparation; the grey, yellow and red boxes refer to the AFM scanned, electrically-written and stress-written regions, respectively (e). SEM image of the prepared lamella with the three regions of interest indicated (f). Scale bars represent 3 µm.

S2 –Additional nano-beam electron diffraction (NBED) data

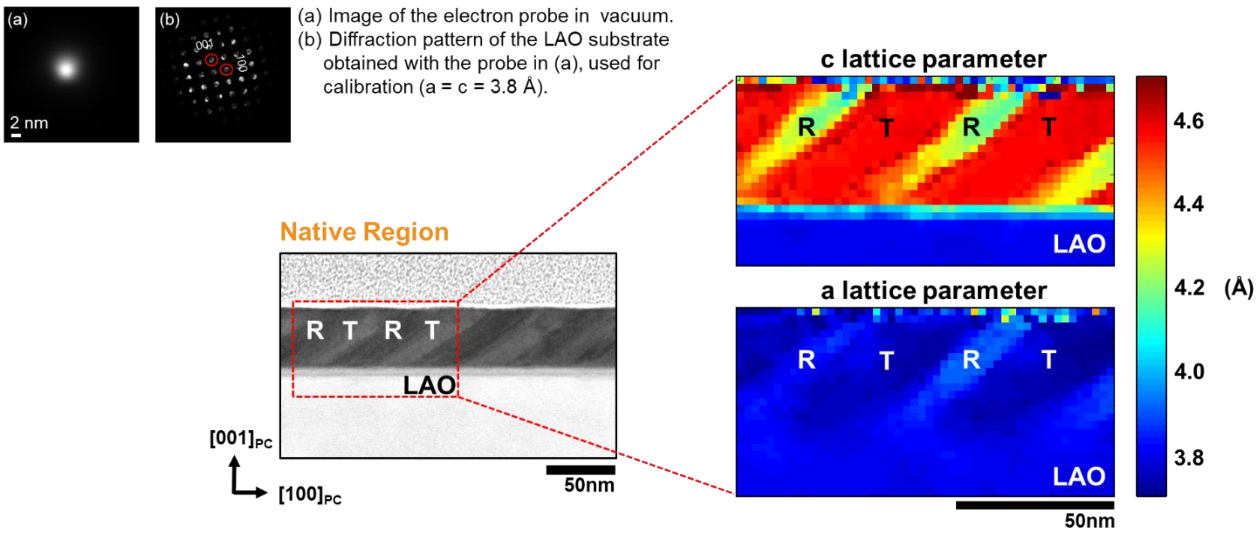

Above: measured out-of-plane and in-plane lattice parameters for a mixed-phase native region. Top left images display the size of the electron probe used for NBED acquisition and an example diffraction pattern of the LAO substrate used for calibration. Our technique was optimised by consulting the work of Rouviere et al. who demonstrate precision in the strain measurement better than 2 x10^{-4} [1].

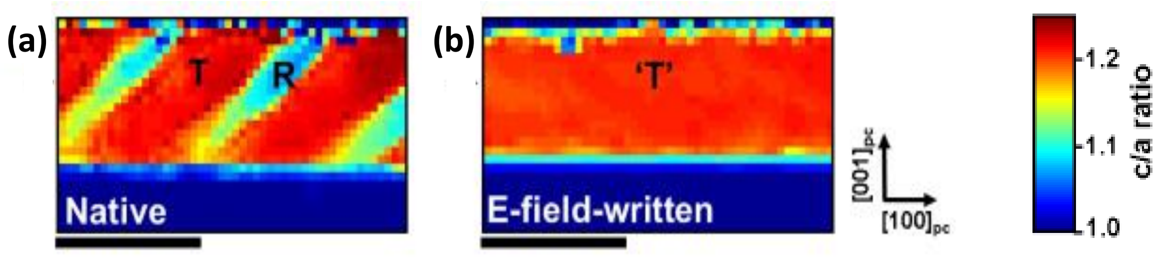

NBED data displaying measured c/a ratios for a native (a) vs. an electrically-written (b) region. The electrically-written T state has a c/a ratio (~1.2) consistent with that of the T matrix in the native mixed-phase region.

S3 – Density of States (DOS) plots

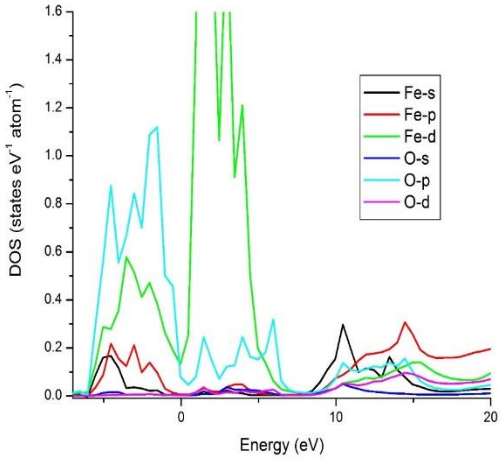

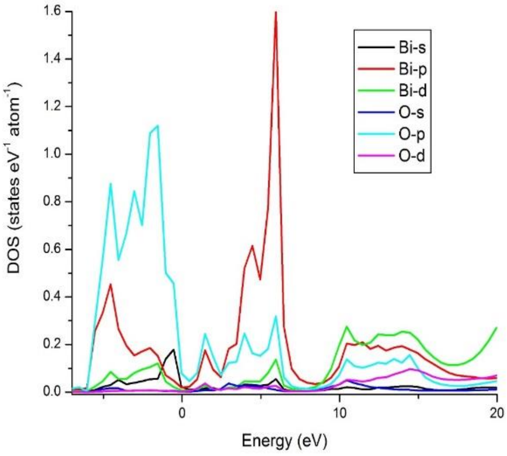

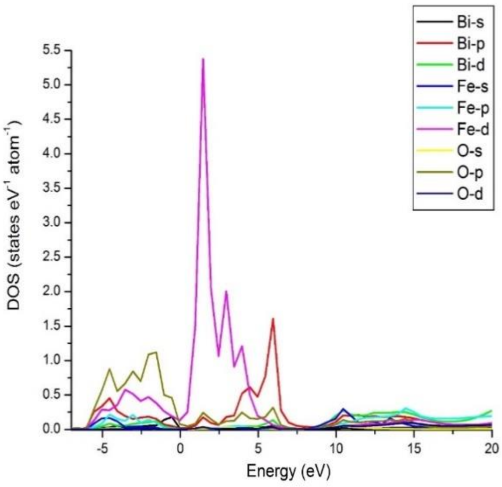

Plots of the density of states (in this example using crystal structure corresponding to the stress-induced R' phase) of Fe-O (top left), Bi-O (top right) and all the elements together (bottom plot) in order to give an idea about the relative contributions of each element to each energy range.

Further details of calculation methods for the EELS edges displayed in Figure 4 of the main paper:

We used multiple-scattering calculations to correlate the Energy Loss Near Edge spectral features in the oxygen and Fe edges with the experiment. They were based in the FEFF9 code [2]. Accurate and well converged muffin-tin potentials and electron densities were calculated in a self-consistent-field (SCF) procedure [3]. The EELS module in FEFF9.6 allows to take into account experimental parameters in EELS such as beam energy, convergence and collection

semiangles and sample to beam orientation [2]. Convergence of the calculations were evaluated by changing the size of the cluster used [4]. The Hedin-Lungqvist self-energy to account for inelastic losses was used. We found that a cluster of about 60 atoms was sufficient to obtain well converged potentials. The UNFREEZEF card was used to include the Bi f states in the SCF calculations typically a cluster of about 220 atoms was enough We have also considered the presence of final-state effects in the observed edges, resulting from the core-level excitation. This core-hole effect is approximated using the RPA approximation.

S4 – Local bond angle measurements

Simultaneous HAADF-STEM and BF-STEM imaging was used to obtain the atomic-column positions of bismuth, iron and oxygen. Figure S4.1 shows a simultaneously acquired z-contrast HAADF image (left) and BF image (right). The field of view for these images (approx. 10 x 10 nm) comprises a single conductive R'-T' phase boundary. In the HAADF image the brightest intensities correspond to Bi atomic columns, the Fe atomic columns appear fainter in intensity. The BF image shows apparent phase contrast localised where the O atoms would be expected, we verify these intensity spots with image simulations presented in Figure S4.2. The high-resolution STEM image simulations were created using the Dr. Probe software [5]. We note that at the studied specimen thickness of 57 ± 3 nm (measured via EELS), the simulated HAADF and BF images match well with the experimental images. We realise that annular bright field (ABF) imaging would allow direct visualisation of both light and heavy atom columns simultaneously with few contrast reversals over a large range of thicknesses, see Figure S4.2. However, we use BF imaging to locate the oxygen atoms, rather than ABF imaging, based on the analysis from Kim et al.'s extensive study [6]. In this study, the authors demonstrate that a notable advantage of BF-STEM imaging comes from the relative insensitivity to the sample mistilt as compared with ABF STEM imaging, enabling better accuracy for locating the oxygen atoms in the BFO structure.

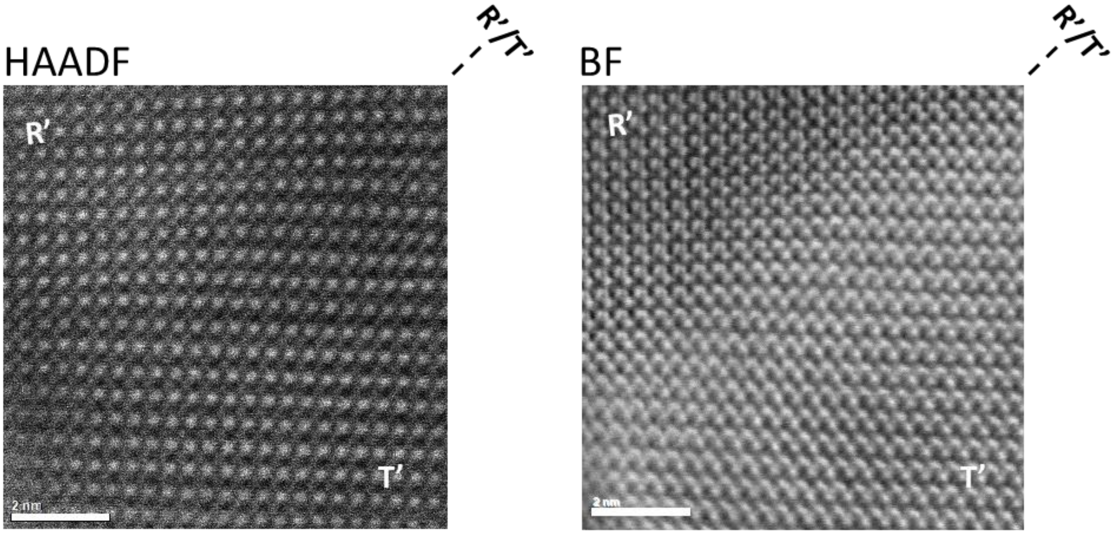

Figure S4.1. Simultaneously acquired HAADF and BF-STEM images of a single conductive R'-T' phase boundary.

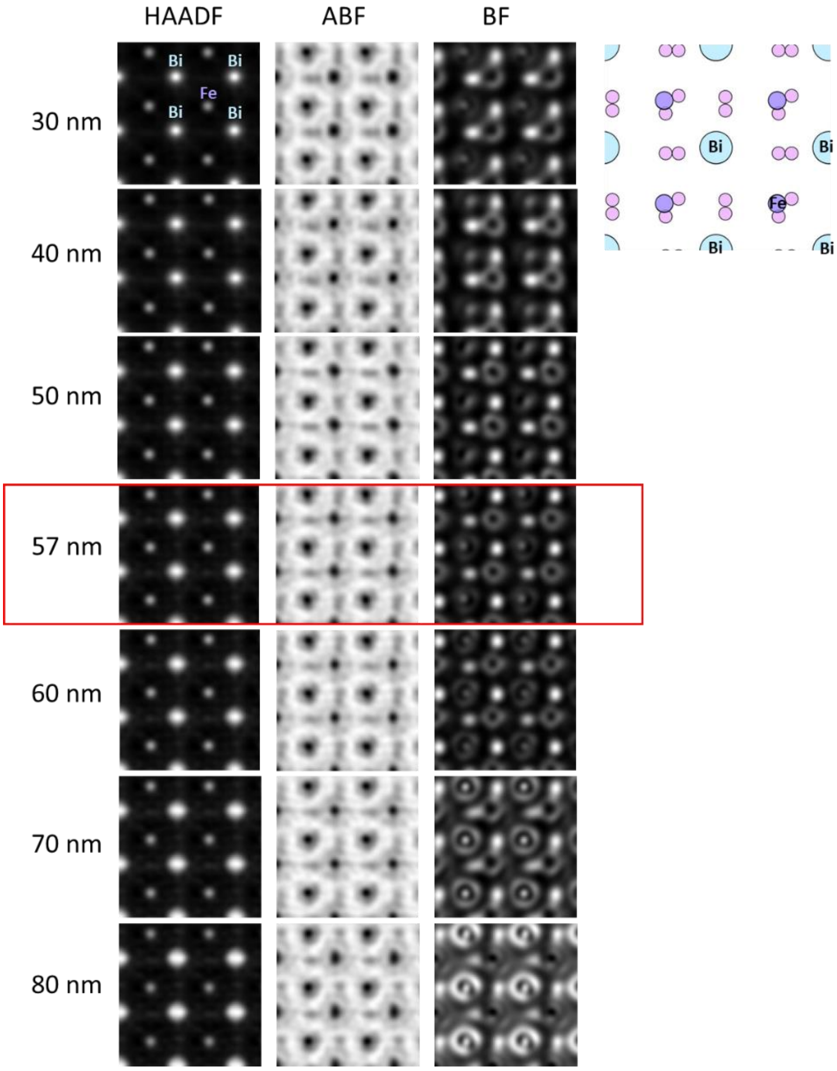

Figure S4.2. Simulated HAADF, BF and ABF STEM images for R' phase $BiFeO_3$ unit cells. The inner and outer detector collection angles for the image simulations were: 70-176 mrad (HAADF), 0-4mrad (BF) and 12-24mrad (ABF).

The experimental atomic positions for Bi, Fe and O were identified from the HAADF and BF STEM images using Atomap [7] which is a python library for analysing atomic resolution transmission electron microscopy images. It relies on fitting 2D Gaussian functions to every atomic column found in an image, and automatically finds all major symmetry axes. In the example of Figure S4.1 the Bi and Fe atomic columns were identified from the HAADF image while the oxygen columns were identified from the BF image. The mutual angles between the A site atom and oxygen (Bi-O-Bi), and the B site atom and oxygen (Fe-O-Fe) were measured and are shown in Figures S4.3 and S4.4 respectively. From this data, we report a possible aggregation of buckled Bi-O-Bi bond angles (red pixels in Figure S4.3) along the conducting R'-T' phase boundary where we have seen extensive elastic distortion (see Figure 5 of main text).

We also observe a trend within the Fe-O-Fe bond angle map of figure S4.4, there appears to be phase contrast between the stress-induced R’ and T’ phases, ~50% of the pixels are blue and ~50% are red, corresponding to approximately straighter Fe-O-Fe bond angles in the R’ unit cells compared to the T’ unit cells of BFO. We conclude by summarising that local bond angle alterations have been induced by the extensive elastic distortion present at these conductive phase boundaries. We emphasise the need for further studies to determine more bond-angle information to better understand the trends linking octahedral bond angle straightening (or buckling) and electrical conductivity at phase boundaries in $BiFeO_3$.

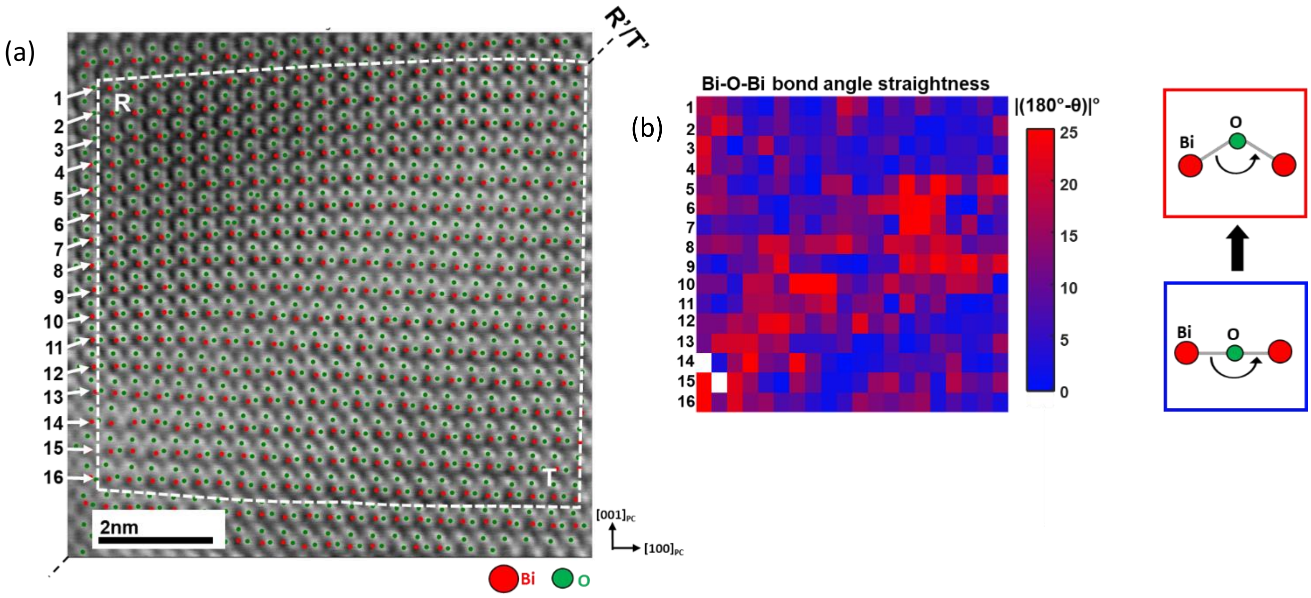

Figure S4.3. Bi-O-Bi bond angles across a stress-induced conductive R’/T’ phase boundary. BF-STEM overview (a) with oxygen (green dot) atom positions and corresponding Bi (red dot) atom positions from HAADF. Map of measured Bi-O-Bi bond angles (b) extracted from the area marked with a white dashed box in (a), showing Bi-O-Bi bond angle buckling associated with the conductive boundary. Schematics of a buckled Bi-O-Bi bond angle and a straightened Bi-O-Bi bond angle are shown on the right. Note, maximum colour bar scale is restricted to 25 degrees.

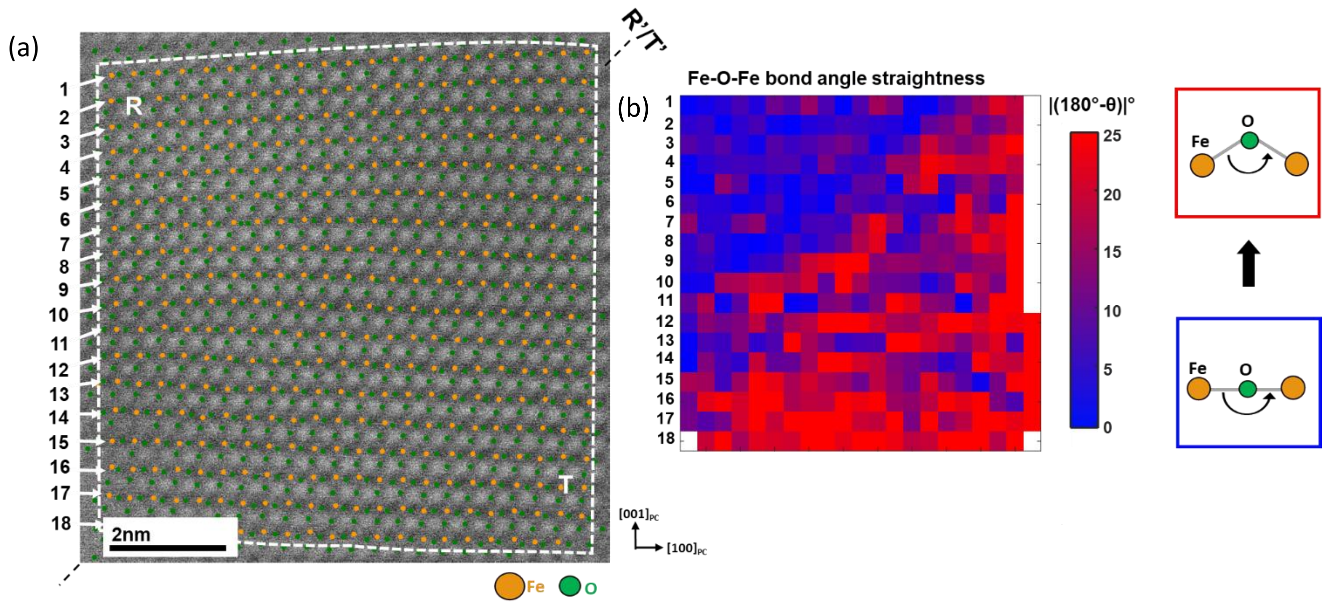

Figure S4.4. Fe-O-Fe bond angles across a stress-induced conductive R'/T' phase boundary. HAADF-STEM overview (a) with Fe (orange dot) atom positions and corresponding oxygen (green dot) atom positions from BF. Map of measured Fe-O-Fe bond angles (b) extracted from the area marked with a white dashed box in (a), showing Fe-O-Fe bond angle phase contrast. Note, maximum colour bar scale is restricted to 25 degrees.

References

[1] J. Rouviere, A. Beche, Y. Martin, T. Denneulin, D. Cooper, *Appl. Phys. Lett.* **2013**, *103*(24), 241913.

[2] J. J. Rehr, J. J. Kas, F. D. Vila, M. P. Prange, K. Jorissen, *Physical Chemistry Chemical Physics.* **2010**, *12*(21), 5503-5513.

[3] B. Ravel, E. A. Stern, R. I. Vedrinskii, V. Kraizman, **1998**, *206*(Ferroelectrics), 407-430.

[4] M. S. Moreno, K. Jorissen, J. J. Rehr, *Micron.* **2007**, *38*(1), 1-11.

[5] J. Barthel, *Ultramicroscopy.* **2018**, *193*, 1-11.

[6] Y. Kim, S. J. Pennycook, A. Y. Borisevich, *Ultramicroscopy.* **2017**, *181*, 1-7.

[7] M. Nord, P. E. Vullum, I. MacLaren, T. Tybell, R. Holmestad, *Advanced Structural and Chemical Imaging.* **2017**, *3*, 9.
